# Proteolytic Activities Expressed by Gastrointestinal Pathogens *Bacillus cereus*, *Listeria monocytogenes* and *Enterococcus faecium* in Different Growth Phases

**DOI:** 10.9734/BMRJ/2015/16402

**Published:** 2015-02-27

**Authors:** Carmen M. Abfalter, Thomas P. Schmidt, Silja Wessler

**Affiliations:** 1 Division of Molecular Biology, Department of Microbiology, Paris-Lodron University, Salzburg, Austria.

**Keywords:** Protease, zymography, Bacillus cereus, Listeria monocytogenes, Enterococcus faecium

## Abstract

**Aims:**

Bacterial proteases are implicated in protein quality control, biofilm formation or might have a direct function in pathogenesis by processing virulence factors or cleaving host factors. In recent years, knowledge of proteases expressed by Gram-negative pathogens remarkably increased. However, investigation of proteases from Gram-positive bacteria is rather rare, but required for the analysis of pathogenesis-relevant proteases. In this study, we extracted and detected proteases from the gastrointestinal pathogens *Bacillus cereus*, *Listeria monocytogenes*, and *Enterococcus faecium* in different growth phases.

**Methodology:**

Bacteria were grown to logarithmic or stationary phases, harvested and extracted by sonication and French press. For the detection of active proteases, zymography analyses were performed using casein and gelatin as substrates to monitor caseinolytic and gelatinolytic activities.

**Results:**

We observed different active proteases with different intensities in bacteria grown to logarithmic or stationary phases. Strong activities as gelatinases were detected in *B. cereus* and distinct caseinolytic proteases exhibiting molecular weights of > 170 kDa, 70 kDa and 45 kDa were shown in *L. monocytogenes* and *E. faecium*, respectively. Interestingly, detected proteases were differentially regulated in bacteria grown to logarithmic or stationary phases.

**Conclusion:**

In summary, the data clearly indicated proteases that are differentially regulated in the Gram-positive pathogens *B. cereus*, *L. monocytogenes*, and *E. faecium*, which might contribute to bacterial pathogenesis.

## 1. INTRODUCTION

Regulated proteases are implicated in many bacterial processes including growth, motility, biofilm formation, modulation of host responses, *etc*. [1]. Investigating pathogens, an increasing number of reports has appeared describing indirect and direct roles of proteases in microbial pathogenesis [2,3]. These proteases exhibit different functions as secreted proteases, in the periplasm of Gram-negative bacteria or in the cytosol of bacteria. However, knowledge of the expression of proteases is still incomplete. In general, approximately 100 different protease families are expressed by eubacteria [4], which can be classified as serine proteases, aspartic proteases, cysteine proteases and metalloproteases dependent on their functional group in the active pocket [5]. Prominent representatives are localized in the bacterial cytosol or periplasm, including the widely expressed Clp (caseinolytic protease), FtsH (filamentous temperature sensitive H), Lon (ATP-dependent protease La), HtrA (high-temperature requirement A), *etc*., which mainly act as degrading proteases in the turnover of intracellular proteins. Functionally, these proteases play important roles in protein quality control, stress tolerance, replication or in the presentation of virulence factors and have often been described as indispensable factors for normal bacterial growth or virulence [6]. Therefore, they have attracted much attention as druggable targets in bacterial virulence in the last years.

In our previous studies, we identified the bacterial ATP-independent chaperone and serine protease HtrA as an active protease expressed in the periplasm of *Helicobacter pylori* (*H. pylori*) and *Campylobacter jejuni* (*C. jejuni*) by casein zymography followed by mass-spectrometry analyses [7,8]. *H. pylori* and *C. jejuni* secrete HtrA that directly interferes with host cell functions by cleaving the cell adhesion protein E-cadherin on epithelial cells. Consequently, HtrA activity allows migration of bacteria across an intact epithelium [7,9] indicating a pivotal role of bacterial serine proteases in infections. The involvement of HtrA in bacterial pathogenesis was also suggested for additional Gram-negative pathogens, such as enteropathogenic *Escherichia coli* (EPEC) and *Shigella flexneri* [10] pointing to the hypothesis that HtrA-mediated cleavage is a prevalent mechanism for gastrointestinal pathogens. Currently, *H. pylori* HtrA serves as a paradigm of a direct protease-mediated interference with pathogenesis of Gram-negative pathogens. Nevertheless, investigation of bacterial proteases is still at the very beginning and the identification of proteolytically active proteins might improve the understanding of complex pathogen-host interactions or tolerance against stress during infection. In particular, proteases expressed by Gram-positive pathogens causing gastrointestinal disorders, such as *Enterococcus faecium* (*E. faecium*), *Listeria monocytogenes* (*L. monocytogenes*) or *Bacillus cereus* (*B. cereus*) have not been investigated yet.

Individual proteases from Gram-negative bacteria are easily detectable by zymography using different substrates, such as gelatin, casein, *etc.* In zymography analyses, proteases can be detected as clear bands in substrate-containing gels after denaturing SDS-PAGE separation under non-reducing conditions, renaturation and protein staining [11]. Therefore, zymography represents a useful technique to detect unknown proteases, which can finally be identified by mass-spectrometry. In this context, disruption of bacteria requires efficient techniques to extract active proteases, which is rather challenging for Gram-positive bacteria and usually needs to be optimized for individual bacterial strains to investigate special biochemical characteristics. In this study, we applied general and simple protocols for French press and sonication to extract proteins exhibiting proteolytic activities from the gastrointestinal pathogens *E. faecium*, *L. monocytogenes* and *B. cereus*. Proteases have been examined for caseinolytic and gelatinolytic activities to obtain more information on the expression of bacterial proteases, which might exhibit important roles in viability and/or pathogenesis.

## 2. METHODOLOGY

### 2.1 Bacterial Strains, Cultivation, and Disruption by Sonication

*L. monocytogenes* (EGD, BUG 600) was cultivated in Luria-Bertani (LB) broth. *B. cereus* (ATCC 14579) was grown in nutrient broth. For *E. faecium* (ATCC 6057) columbia agar containing 5% sheep blood (BD BBL, Heidelberg, Germany) or brain-heart-infusion (BHI) medium (Carl Roth, Karlsruhe, Germany) were used. All bacteria strains (Table 1) were cultivated at 37°C. For protein extraction of bacteria grown in the logarithmic and stationary growth phase, bacteria were cultured in broth overnight (stationary phase). The next day, bacterial culture was 1:20 diluted in fresh medium and grown at 37°C to an OD_600_ of approximately 0.8 (logarithmic phase). Bacterial cultures were centrifuged at 4500 × g for 20 min at 4°C. Pelleted bacteria were then harvested in PBS (Sigma, Vienna, Austria) or lysis buffer (20 mM Tris pH 7.5, 100 mM NaCl, 1 % Triton X-100, 0.5 % DOC, 0.1 % SDS, 0.5 % NP-40). Where indicated 5 μg/ml lysozyme (Lactan, Graz, Austria) was added for 1 h at 37°C. Bacteria suspended in PBS or lysis buffer were sonicated on ice 3 times for 45 sec with 50 % power. All bacterial lysates were centrifuged at 16000 × g for 20 min at 4°C. The protein content of the lysates was measured using Bradford protein assay (Carl Roth, Karlsruhe, Germany). All experiments were repeated at least four times.

### 2.2 Disruption by French press

For the disruption of bacteria by French pressure cell press (French press, SLM Aminco, Spectronic Instruments, USA), equal numbers of bacteria were harvested in PBS containing 5 μg/ml lysozyme as indicated. A volume of 10 ml suspension was filled in a 20k Manual-Fill cell. The French press was operating with 11000 psi as maximal pressure. Two cycles were performed for every sample.

### 2.3 Non-reducing Sodium Dodecyl Sulfate Polyacrylamide Gel Electrophoresis (SDS-PAGE)

Protein lysates were separated using SDS-PAGE. All samples were incubated with non-reducing sample buffer (125 mM Tris-HCl (pH 6.8), 20% glycerin, 4% SDS, 0.02% bromophenol blue) for 20 min at room temperature. 10 μg protein of all samples were loaded onto the gels. Gels were stained with Coomassie Blue G250 (BioRad, Vienna, Austria).

### 2.4 Zymography

5 μg (*B. cereus*), 10 μg (*E. faecium*) or 20 μg (*L. monocytogenes*) protein was analyzed by zymography using 0.1 % casein or 0.1 % gelatin as substrates under non-reducing conditions as described before [8]. The gel was then renatured in 2.5 % Triton X-100 twice for at least 1 hour. Afterwards it was incubated in developing buffer (50 mM Tris pH 7.4, 200 mM NaCl, 5 mM CaCl2, 0.02 % Brij35) for at least 24 hours at 37°C. After staining the gel overnight using 0.5 % Coomassie Blue R250 (BioRad, Vienna, Austria) proteolytic activities were indicated by transparent bands. Transparent bands were finally quantified using the ImageLab software (BioRad, Vienna, Austria). Graphical presentation and statistical evaluations (student t-test) were performed with GraphPad Prism 5. P values = ***p ≤ 0.001, **p ≤ 0.01 and *p ≤ 0.05 were considered statistically significant.

## 3. RESULTS

**Fig. 1:**
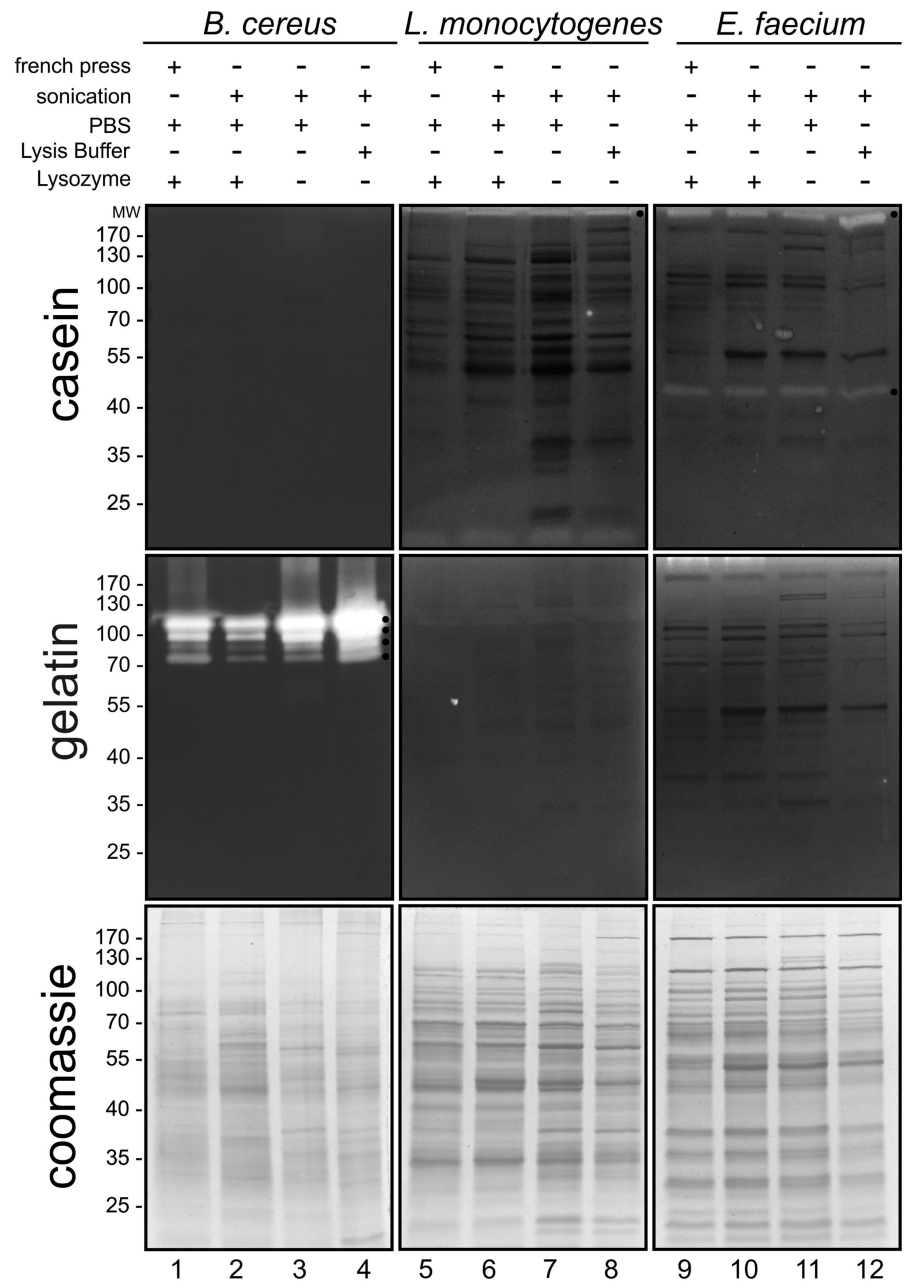
Detection of caseinolytic and gelatinolytic activities in Gram-positive bacteria

Investigation of caseinolytic proteases of Gram-negative pathogens, such as *H. pylori*, *C. jejuni*, *Shigella flexneri* or EPEC by zymography is well established [7,8,10]. Here, we aimed at an analysis of the differentially regulated expression of active proteases by Gram-positive pathogens. Because efficient disruption of Gram-positive bacteria might also be dependent on the tested microbial strain, different extraction methods for *B. cereus*, *L. monocytogenes* and *E. faecium* were applied, followed by the detection of proteolytic activities in zymogram analyses using casein or gelatin as substrates (Fig. 1). Proteins from bacteria were extracted in PBS containing lysozyme by French press (Fig. 1, lanes 1, 5, 9), sonication in PBS containing lysozyme (Fig. 1, lanes 2, 6, 10), sonication in PBS (Fig. 1, lanes 3, 7, 11), and sonication in lysis buffer (Fig. 1, lanes 4, 8, 12). Without further strain-specific optimizations, disruption of bacteria by French press and sonication in PBS resulted in an enrichment of cytoplasmic proteins, while sonication in lysis buffer containing nonionic and anionic detergents lysed bacteria and increased solubility of membrane proteins. Equal protein amounts were then analyzed by casein zymography or gelatin zymography and coomassie staining.

Lysates of *B. cereus* showed strong proteolytic activities using gelatin as a substrate. Four protease variants were detected exhibiting a molecular weight ranging from ~70 to ~125 kDa, whereas no activity was observed using casein as a substrate (Fig. 1, lanes 1-4, black asterisks). As a control for protein loading in zymogram analysis, equal protein amounts were separated by SDS PAGE under non-reducing conditions (Fig. 1, lanes 1-4). In contrast to *B. cereus*, a relatively weak activity of a high molecular weight caseinolytic protease (> 170 kDa) from *L. monocytogenes* was detected (Fig. 1, lanes 5-8, black asterisk), which was less obvious in extracts obtained by the French press. However, a gelatinolytic protease from *L. monocytogenes* has not been detected under these conditions (Fig. 1, lanes 5-8). Similar observations were made in *E. faecium*. In *E. faecium* lysates obtained by all tested methods, casein-degrading activities of > 170 kDa and about 45 kDa were observed, but no gelatin-targeting proteases (Fig. 1, lanes 9-12, black asterisks).

**Fig. 2:**
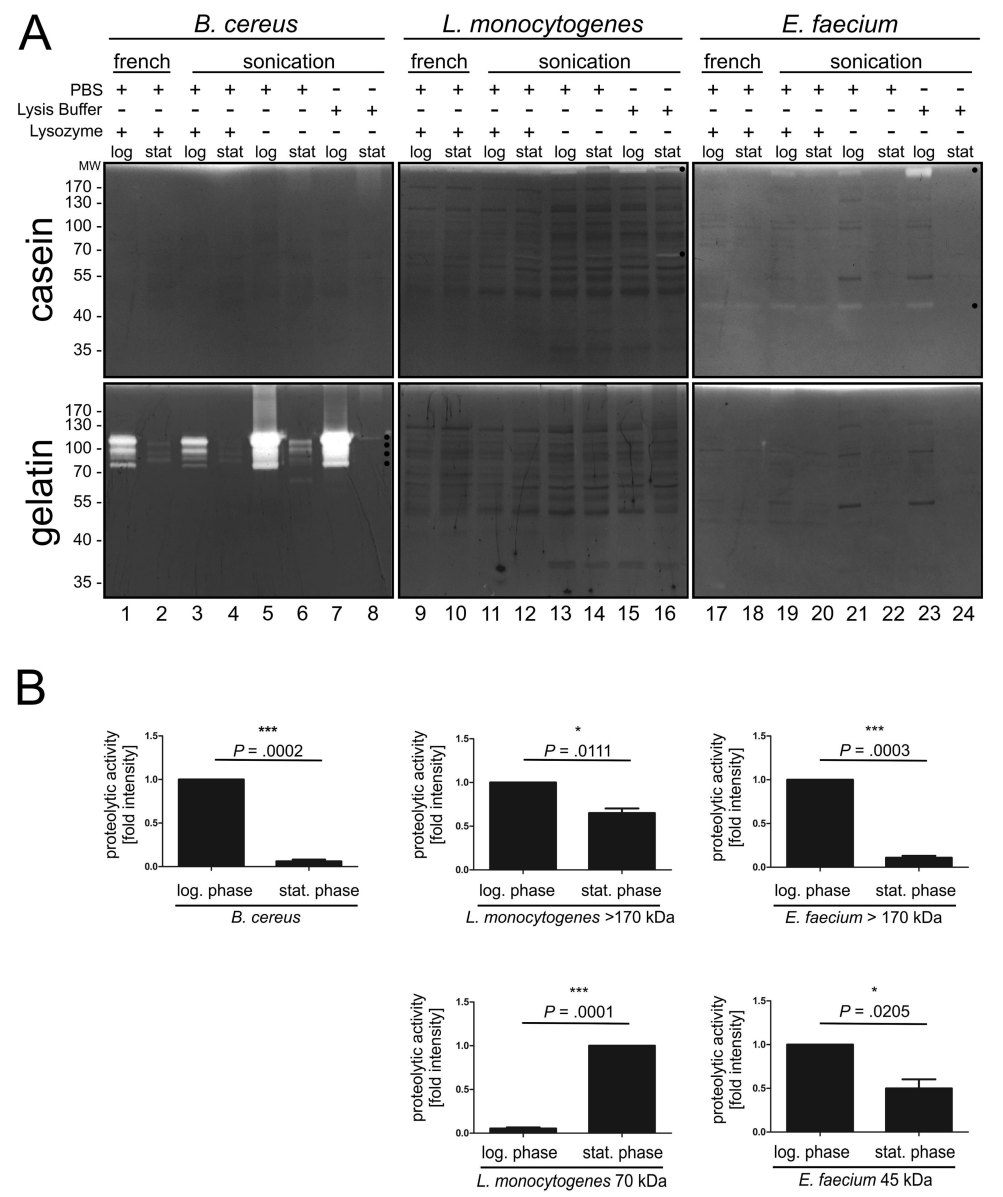
Detection of proteases of Gram-positive bacteria grown to different growth phases

Expression of proteases and regulated proteolysis can also depend on bacterial growth phases allowing specific adaptions of bacteria to a changing environment [12] and bacterial development [13]. Therefore, we analyzed the activity of detected proteases in pathogens in different growth phases corresponding to growing bacteria in the logarithmic phase and starving bacteria in the stationary phase. Equal numbers of bacteria were lysed as indicated and analyzed by zymography (Fig. 2A) and SDS PAGE under non-reducing conditions (data not shown). Changes in proteolytic activities (black asterisks) were quantitated and presented in bar diagrams (Fig. 2B). The data represent the mean ± S.D. calculated from three independent experiments as x-fold intensity. The proteolytic intensities in extracts from bacteria grown to the logarithmic phase were set as 1 and compared to the corresponding activities of bacteria grown to the stationary phase. In contrast, the activity of the 70 kDa protease of *L. monocytogenes* grown to the stationary phase was set as 1 and compared with the corresponding activity of bacteria in the logarithmic phase. The four gelatinolytic activities from *B. cereus* were collectively quantified. For the quantification protease activities of *B. cereus* and *E. faecium*, extracts obtained by French press were included, while caseinolytic activities in lysis buffer-extracted *L. monocytogenes* were quantified. As previously indicated, no caseinolytic proteases were detected in *B. cereus* in the logarithmic phase as well as at the stationary phase (Fig. 2A, lanes 1-8). Interestingly, the expression or activity of observed gelatinases was drastically downregulated in *B. cereus* grown to the stationary phase (Fig. 2A, lanes 1-8). Quantification of proteolytic activities confirmed this observation (Fig. 2B). The gelatinase of *B. cereus* was more than 90% down-regulated in the bacteria grown to the stationary phase compared to bacteria in the logarithmic growth phase (Fig. 2B, left graphics). An opposite effect was observed in *L. monocytogenes*. Here, a casein-degrading protease of approximately 70 kDa was clearly detectable in bacteria grown to the stationary phase, which were extracted by sonication in lysis buffer. This protease was less visible after disruption of bacterial cultures in the stationary phase by French press or sonication in PBS without detergents (Fig. 2A, lanes 9-16). On the other hand, the high molecular weight protease of > 170 kDa, which was slightly visible in bacteria grown to the logarithmic phase decreased in bacteria in the stationary phase (Fig. 2A, lanes 9-16). Quantification of caseinolytic activities revealed a decrease of approximately 35% of the > 170 kDa protease and an approximate 20-fold increase of the 70 kDa caseinase (Fig. 2B, middle graphics). Finally, lysates of *E. faecium* contained differentially regulated proteases as shown in the casein zymogram. Both, the ~45 kDa and >170 kDa protease were active in bacteria in the logarithmic phase, but significantly decreased in the stationary phase. Obviously, *E. faecium* in the stationary phase were only poorly disrupted by sonication, but equally efficient by the French press method as implied by the background detection of proteins (Fig. 2A, lanes 17-24) and by coomassie-stained proteins in SDS PAGEs as loading controls (data not shown). Therefore, quantification of these proteolytic activities was performed for bacteria extracted by the French press method, which resulted in equable disruption of *E. faecium*. Here, a 90% and a 50% reduction were detected for the > 170 kDa and the 45 kDa activities, respectively (Fig. 2B, right graphics). In conclusion, these data indicate that Gram-positive pathogens express and/or regulate proteases dependent on their growth phase.

## 4. DISCUSSION

In the last years, proteases originating from bacterial pathogens received increased attention as it became clear that proteases are implicated in pathogenesis via direct and indirect mechanisms. The majority of studies was performed in Gram-negative pathogens; hence, it was tempting to analyze proteases expressed by Gram-positive pathogens by zymography. We used different methods for efficient extraction of proteases. In fact, sonication in nonionic and anionic detergent-containing lysis buffer was a simple and rapid method for bacterial disruption leading to an enhanced solubility of cytoplasmic and membrane-located proteases. Generally, proteases belong to a large and very complex group of enzymes that considerably differ in structure, substrates, and catalytic mechanism. In *B. cereus*, *L. monocytogenes* and *E. faecium*, we detected caseinolytic or gelatinolytic proteases, respectively. Nevertheless, information on those active proteases in Gram-positive bacteria is rare. An unidentified 105 kDa collagenase with unknown function was described in 1999, which exhibited similarities with clostridial collagenases [14]. Bacterial collagenases can be implicated in the destruction of tissue [15] and are considered as important virulence factors. However, whether the 105 kDa collagenase corresponds to one of the proteolytic activities detected in gelatin zymography in our report remained unknown. The situation is not different for *L. monocytogenes*. Here, the 26 kDa serine protease ClpP has been described as an essential factor for the intracellular life of *L. monocytogenes in vivo* [16] and it was hypothesized that ClpP enhances listerial survival under stress conditions. During infection, autoprocessing of a listerial metalloprotease (MP1) was important in bacterial phosphatidylcholine phospholipase C (PC-PLC) maturation and intracellular life cycle [17,18]. As another additional protease, HtrA appears to play a role in *L. monocytogenes* pathogenesis through enhancing its viability under stress conditions [19,20]. In Enterococci, a gelatinase was reported, which is implicated in biofilm formation [21]. Although the activity of these proteases was not demonstrated in direct assays using purified enzymes, bacterial knock-out mutants implied their functional roles. Therefore, these observations are interesting since we directly detected several activities in *E. faecium*, *L. monocytogene*s and *B. cereus*, which were differentially regulated in the logarithmic and stationary phase indicating that regulated protease activity is crucially important in a dynamic environment [12]. The strong activity of the *B. cereus* gelatinase was decreased in bacteria grown to the stationary phase. In *L. monocytogenes*, the high molecular weight caseinolytic protease decreased while a ~70 kDa protease activity strongly increased. In *E. faecium*, both, the > 170 kDa and ~40 kDa activity was drastically down-regulated in bacteria grown to the stationary phase. This might reflect changing conditions for bacteria requiring enhanced degradation of misfolded protein during protein quality control. In fact, an *htrA* knock-out mutant of *L. monocytogenes* showed an impaired biofilm formation supporting the hypothesis that proteases are implicated in bacterial adaptation and development [20]. In *E. coli*, proteolytic heat shock proteins were differentially regulated via an alternative sigma factor [22] allowing the bacteria to reprogram global transcription rapidly and to adapt to changes in the environment. Dependent on the growth phase of *L. monocytogenes*, differentially expressed proteins, including the protease ClpB, have already been identified [23,24]. For *B. cereus* and *E. faecium*, those studies are not available yet, but our data imply that proteases are differentially regulated in these pathogens.

## 5. CONCLUSION

In this study, we detected proteolytic proteins expressed by the gastrointestinal pathogens *B. cereus*, *L. monocytogenes* and *E. faecium* in zymography analyses. Active proteases degraded casein or gelatin as substrates and were differentially expressed or regulated in growing and starving bacteria. Although the proteases were not identified yet, we assume that they might play a functional role in bacterial survival, biofilm formation or pathogenesis by targeting intracellular misfolded proteins, and/or extracellular matrix proteins. Because *B. cereus*, *L. monocytogenes*, and *E. faecium* are disease-relevant pathogens of the gastrointestinal tract, we assume that improved knowledge of stress-and/or density-regulated protease activity could also be important in clinical practice. Further research is necessary to characterize these proteases as putative pathogenic or virulence factors.

## Figures and Tables

**Table 1:** Bacterial strains used in this study

Bacteria	Strain	Disease
*Bacillus cereus*	ATCC^1^ 14579	Food-borne nausea, vomiting, diarrhea
*Listeria monocytogenes*	EGD, BUG 600	Listeriosis
*Enterococcus faecium*	ATCC 6057	Urinary tract, wound, and soft tissue infections in immunocompromised patients, can occur as a commensal in the intestine

1 ATCC, American Type Culture Collection (www.atcc.org).
